# Supplementary material for: Involvement of MicroRNA-27a-3p in the Licorice-Induced Alteration of Cd28 Expression in Mice
Source: Genes (Basel). 2022 Jun 25;13(7):1143. doi: 10.3390/genes13071143 (PMC9317804; doi:10.3390/genes13071143)
Supplement: Supplementary file 1 [file genes-13-01143-s001.zip › Supplementary Figures.pdf]

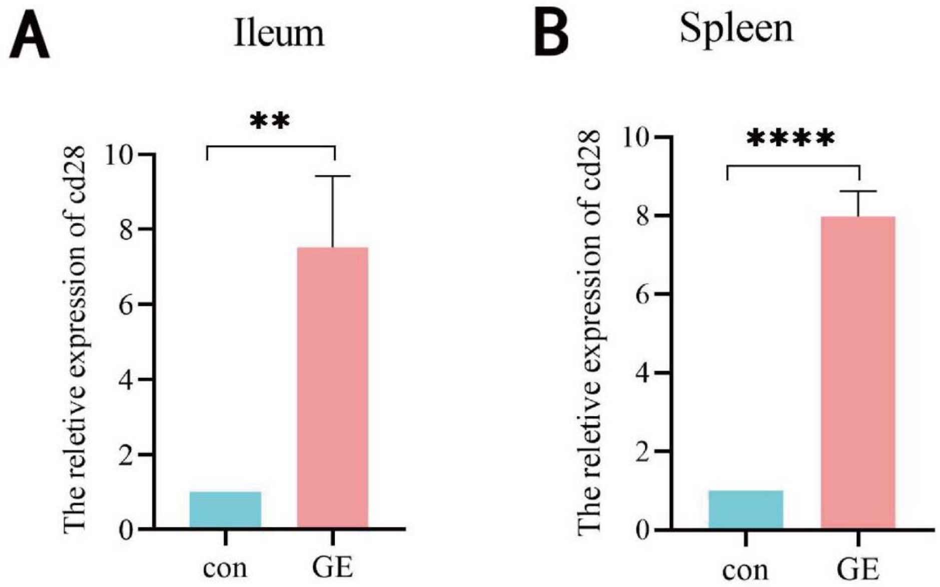

Figure S1. qRT-PCR detection of *Cd28* in healthy mice treated with licorice decoction. (**A**) The expression of *Cd28* gene in the ileum. (**B**) The expression of *Cd28* gene in the spleen. con, control group; GE, licorice decoction treatment group. **, $p < 0.01$; ***, $p < 0.001$; ****, $p < 0.0001$.

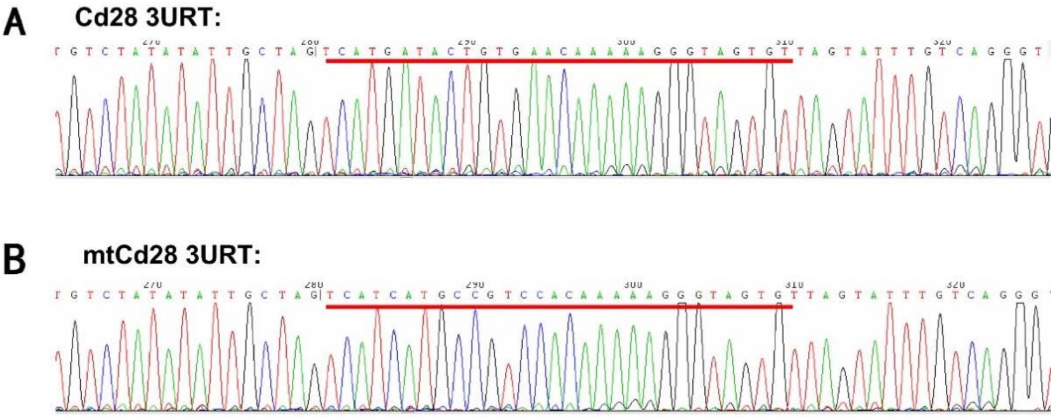

Figure S2. Sequencing results of the recombinant dual-luciferase reporter vectors. (**A**) The sequence of wild-type *Cd28* 3′UTR in psiCHECK-2. (**B**) The sequence of mutant *Cd28* 3′UTR in psiCHECK-2.
