# Supplementary material for: Involvement of MicroRNA-27a-3p in the Licorice-Induced Alteration of Cd28 Expression in Mice
Source: Genes (Basel). 2022 Jun 25;13(7):1143. doi: 10.3390/genes13071143 (PMC9317804; doi:10.3390/genes13071143)
Supplement: Supplementary file 1 [file genes-13-01143-s001.zip › Supplementary Tables.pdf]

Table S1. Primers used for vector construction.

Primers	Primer Sequences (5′-3′)
Cd28-3UTR-*U*	ccgCTCGAGCCAAGAAGAATGTGAGAGGAAGTA
Cd28-3UTR-*D*	ataagaatGCGGCCGCAGCAGCTGAGCCCACAAAGTT
For-mut	TCATCATGCCGTCCACAAAAAGGGTAGTGT
Rev-mut	GTGGACGGCATG ATGACTAGCAATATATAGAC

Note: the underlined section is the restriction enzyme digestion site.

Table S2. Primers used for qRT-PCR.

Primers	Primer Sequences (5′-3′)
Cd28-U-rt	CTGGTCGTGGTTGCTGGAGTC
Cd28-D-rt	AGGGGCGTAGGGCTGGTAAG

Table S3. The hub genes involved in the miRNA–mRNA network.

Node	MCC
Cd28	6
Lmo3	5
Igf1	5
Sox5	5
Sh3kbp1	5
Limd2	5
Gpr174	5
Slitrk2	4
Runx1t1	4
Epha5	4
AI593442	4
Ipcef1	4
Ypel2	4
Tnfrsf26	4
St8sia4	4
Sh2d3c	4
Scai	4
Rnf144a	4
Mef2c	4
Lpl	4
Itpripl1	4
Inpp4b	4
Cxcl12	4
Cecr2	4
Ar	4
Scn9a	3
Rimklb	3
Hlf	3
Gpr85	3
Usp2	3
St8sia3	3
Rgs7bp	3
Rbfox3	3
Kcnc1	3
Tbc1d4	3
Phlda3	3
Tmem154	3
Lca5	3
Stx11	3

Slc14a1	3
Pi15	3
Lonrf3	3
Itga4	3
B3galt2	3
Adam12	3
Xkrx	3
Snn	3
Slc43a1	3
Rrad	3
Ptges	3
Plxnc1	3
Pcdh15	3
Lrrc32	3
Lpin1	3
Irf4	3
Il2ra	3
Il10ra	3
Fasn	3
Far1	3
Edaradd	3
Ebf1	3
Chst11	3
Cdc6	3
Adamts10	3

Table S4. The DEgenes involved in the three enriched KEGG pathways.

Pathways	Genes
	Cd86
	Itga4
	Mpz
Cell adhesion molecules	*Nrcam*
	Nrxn2
	Pecam1
	Spn
↑	*Cd28*
overlap	*Cd4*
↓	*Ptprc*
T cell receptor signaling pathway	*Grap2*
	Lck
	Cd247
	Vav1
	Ccl21a
Viral protein interaction with cytokine and cytokine receptor	*Cxcl12*
	Cxcl13
	Cxcr3
	Il10ra
	Il18
	Il2ra

Table S5. The DEmiRNAs involved in regulating the 21 DEgenes.

miRNAs	Connections
mmu-miR-24-3p	9
mmu-miR-328-3p	8
mmu-miR-128-3p	4
mmu-miR-27a-3p	3
mmu-miR-142a-5p	3

Table S6. The DEmiRNAs with screening criteria |log(fold difference)| ≥ 2, $p \le 0.01$.

miRNAs	logFC	*p* Value
mmu-miR-183-5p	7.633256651	1.66×10^{-162}
mmu-miR-182-5p	7.419807229	1.84×10^{-158}
mmu-miR-129-5p	6.368284928	1.15×10^{-127}
mmu-miR-184-3p	2.243903238	2.65×10^{-25}
mmu-miR-27a-3p	−2.043916623	1.54×10^{-21}

Table S7. The target genes of mmu-miRNA-27a-3p among 271 common genes.

Genes
Cd28
Cxcl12
Grap2
A630001G21Rik
Abcg3
Adamts10
Angpt2
Ankrd44
Ar
Arhgap15
Cd5
Cecr2
Cep41
Cklf
Fam13a
Fasn
Gja5
Glipr2
Gpr174
Hephl1
Igf1
Ikzf1
Inpp4b
Ipcef1
Irf4
Itpripl1
Klhl4
Lca5
Limd2
Lonrf3
Loxhd1
Lpin1
Lpl
Mef2c
Nat8l
Pabpc4l
Paqr9
Phlda3
Pi15
Pik3cg
Plek
Prkcb
Ptges
Rasal3
Ripor2
Rnf144a
S1pr2

Scai
Sh2d3c
Sh3kbp1
Slc14a1
Slc16a9
Slc4a11
Slc7a10
Snn
Snx22
Sorl1
Sox5
Spn
St8sia4
Tbc1d4
Tenm4
Tnfrsf26
Trim30d
Xkrx
Ypel2
Zfp831
